# Interpreting results, impacts and implications from WHO FCTC tobacco control investment cases in 21 low-income and middle-income countries

**DOI:** 10.1136/tc-2023-058337

**Published:** 2024-05-02

**Authors:** Nathan Mann, Garrison Spencer, Brian Hutchinson, Carrie Ngongo, Dudley Tarlton, Douglas Webb, Daniel Grafton, Rachel Nugent

**Affiliations:** 1 RTI International, Research Triangle Park, North Carolina, USA; 2 United Nations Development Programme, Istanbul, Turkey; 3 United Nations Development Programme, Amman, Jordan; 4 Department of Global Health, University of Washington, Seattle, Washington, USA

**Keywords:** Tobacco control, Low/Middle income country, Policy, Advocacy

## Abstract

**Background:**

Tobacco control investment cases analyse the health and socioeconomic costs of tobacco use and the benefits that can be achieved from implementing measures outlined in the WHO Framework Convention on Tobacco Control (WHO FCTC). They are intended to provide policy-makers and other stakeholders with country-level evidence that is relevant, useful and responsive to national priorities and policy context.

**Methods:**

This paper synthesises findings from investment cases conducted in Armenia, Cabo Verde, Cambodia, Chad, Colombia, Costa Rica, El Salvador, Eswatini, Georgia, Ghana, Jordan, Laos, Madagascar, Myanmar, Nepal, Samoa, Sierra Leone, Sri Lanka, Suriname, Tunisia and Zambia. We examine annual socioeconomic costs associated with tobacco use, focusing on smoking-related healthcare expenditures, the value of lives lost due to tobacco-related mortality and workplace productivity losses due to smoking. We explore potential benefits associated with WHO FCTC tobacco demand-reduction measures.

**Results:**

Tobacco use results in average annual socioeconomic losses of US$95 million, US$610 million and US$1.6 billion among the low-income (n=3), lower-middle-income (n=12) and upper-middle-income countries (n=6) included in this analysis, respectively. These losses are equal to 1.1%, 1.8% and 2.9% of average annual national gross domestic product, respectively. Implementation and enforcement of WHO FCTC tobacco demand-reduction measures would lead to reduced tobacco use, fewer tobacco-related deaths and reduced socioeconomic losses.

**Conclusions:**

WHO FCTC tobacco control measures would provide a positive return on investment in every country analysed.

**WHAT IS ALREADY KNOWN ON THIS TOPIC**

- Tobacco use has negative effects on both health and sustainable development. Prior studies of the impact of tobacco use demonstrate the economic burden associated with tobacco use. Previous studies have primarily been in high-income countries.

**WHAT THIS STUDY ADDS**

- This study summarises results and synthesises findings from WHO Framework Convention on Tobacco Control country-specific tobacco control investment cases conducted for 21 diverse low-income and middle-income countries, representing various geographies and populations.

**HOW THIS STUDY MIGHT AFFECT RESEARCH, PRACTICE OR POLICY**

- Despite varying country contexts, results from this study demonstrate that the health and economic benefits of investments in effective tobacco control greatly outweigh the costs of implementing such measures.

## Introduction

Tobacco use results in premature death, illness, increased healthcare costs and economic losses.^1^ Numerous diseases, such as cancer, cardiovascular disease and lung disease, are caused by tobacco use.^2^ The life expectancy for people who smoke is up to 10 years shorter than people who do not smoke.^3^ Tobacco use harms people who consume tobacco products as well as people who are exposed to secondhand smoke, which includes children and other vulnerable populations.^4^ Premature mortality due to tobacco use results in both societal losses and the loss of human capital. The health consequences associated with tobacco use result in increased healthcare expenditures as well as diminished worker productivity.^5–7^ Tobacco use is also associated with increased poverty and socioeconomic disparities.^8–11^

The WHO Framework Convention on Tobacco Control (WHO FCTC) is an international treaty that entered into force in 2005. The 182 parties to the WHO FCTC have committed to implementing evidence-based tobacco control efforts, including both tobacco demand-reduction and supply-reduction measures. Nearly all WHO FCTC Parties have implemented at least some of the measures required by the WHO FCTC. However, most have not achieved full implementation.^12^

The WHO FCTC Secretariat launched the FCTC 2030 project in 2016 to provide technical assistance and support to governments in low-income and middle-income countries wishing to implement evidence-based tobacco control efforts as required by the WHO FCTC.^13 14^ All parties to the WHO FCTC who are eligible to receive official development assistance from the Organisation for Economic Co-operation and Development and who show political commitment to advancing implementation of WHO FCTC measures are eligible to join the FCTC 2030 project through an open application process. Participating countries have been selected based on their commitment and readiness to advance WHO FCTC implementation through multisectoral action and intention to strengthen tobacco taxation, their interest in receiving technical assistance and through participation in a WHO FCTC needs assessment.^15^

The negative health consequences of smoking have been well established since the early 1960s.^16 17^ Previous studies have also estimated the global economic impacts of tobacco use.^18–21^ Goodchild *et al* 21 estimated that smoking-related healthcare costs in 2012 equalled US$422 billion (5.7% of global healthcare expenditures). Including productivity losses, the total economic impact jumps to US$1.4 trillion, which is equivalent to 1.8% of the world’s gross domestic product (GDP).^21^ Previous studies of the health and economic consequences of tobacco use have mostly been in high-income countries.^21 22^

Countries participating in the FCTC 2030 project are eligible to receive a country-specific tobacco control investment case to help select and justify national investments in tobacco control. The investment cases quantify the current health and socio-economic losses caused by tobacco use and evaluate the future costs and benefits of implementing evidence-based tobacco control measures as required under the WHO FCTC.^23–25^ With support from the WHO FCTC Secretariat and the UK government, the research institute RTI International, in collaboration with the United Nations Development Programme and the WHO FCTC Secretariat, prepared country-specific tobacco control investment cases.

Between 2017 and 2022, WHO FCTC national tobacco control investment cases were completed for a total of 21 LMICs as part of the FCTC 2030 project.^24^ This paper presents and summarises results and findings from those investment cases and synthesises those results to identify patterns and insights. Investment cases completed later are not included in this synthesis. We examine differences across countries related to population characteristics, smoking prevalence, the current health and socio-economic burden of tobacco use, and the forecasted impacts of implementing evidence-based tobacco demand-reduction measures required under the WHO FCTC. For this analysis, we group countries by country income—low-income countries (LICs), lower-middle-income countries (LMICs) and upper-middle-income countries (UMICs).

This paper is part of a collection of papers, published together as a supplement, describing WHO FCTC tobacco control investment cases conducted for 21 low-income and middle-income countries between 2017 and 2022. Other papers in the supplement that this paper is included in provide additional information about the WHO FCTC and the FCTC 2030 project,^24^ as well as the data and methods used to conduct WHO FCTC tobacco control investment cases from 2017 to 2022.^25^ Information regarding the WHO FCTC tobacco control investment case methodology has previously been summarised in a published article synthesising results from four countries in the Americas.^26^ Additional details regarding the data sources and methods for WHO FCTC tobacco control investment cases can also be found as part of country-specific investment case reports available online.^27^

## Methods

### Country-specific WHO FCTC tobacco control investment cases

The WHO FCTC tobacco control investment cases use a prevalence-based cost-of-illness approach to estimate the current (ie, 1 year) health and socio-economic losses associated with tobacco use. Country-specific estimates of annual total and tobacco-related mortality and morbidity, by sex and 5-year age group, for diseases causally linked with tobacco use were obtained from the Global Burden of Disease study.^28^ Using those estimates, a population-attributable fraction (PAF) was derived for each sex, 5-year age group and disease. Economic losses include direct costs from smoking-related healthcare expenditures as well as the monetised value of lost workplace productivity due to smoking. Societal losses include the monetised value of years of life lost due to premature mortality due to tobacco use. Together we term these ‘socio-economic’ losses.

Goodchild *et al* estimated the smoking-attributable fraction (SAF) of healthcare expenditures in 2012 for 152 countries.^21^ Country-specific SAF estimates for healthcare expenditures were based on results from Goodchild *et al* with adjustments or recalculations made for certain countries. Smoking-attributable healthcare expenditures were estimated by multiplying total national healthcare expenditures^29^ by the SAF of healthcare expenditures obtained or derived for each country. To place a value on deaths caused by tobacco use, years of life lost due to tobacco-attributable mortality were monetised using established economic valuation methods.^25^ The economic value associated with workplace productivity losses due to smoking was estimated based on published estimates of lost work time due to absenteeism (additional days of work missed per year due to smoking-related illnesses), presenteeism (reduction in productivity due to smoking-related causes) and unsanctioned smoking breaks (additional time in breaks from work beyond what is allowed by law or employer). Parameter estimates for the amount of lost work time per person who smokes due to absenteeism, presenteeism and excess smoking breaks per employed worker who smokes came from previously published literature.^5–7^ Lost productivity was calculated by multiplying the average lost work time per person who smokes by the estimated number of employed adults who smoke by average wage rates in countries.^25^ Adult smoking rates for these calculations were obtained from national data sources provided by country partners. In cases when country partners were unable to provide those data, country-specific adult smoking prevalence was obtained from the country profile from the most recent version of the WHO report on the global tobacco epidemic available at the time when the tobacco control investment case was conducted. A summary of model inputs for each country is available in online supplemental table 1.

10.1136/tc-2023-058337.supp1 Supplementary data

After estimating the current health and socio-economic costs associated with tobacco use, the WHO FCTC tobacco control investment cases evaluated the potential impact of implementing or expanding key tobacco demand-reduction measures—which have been agreed to by Parties of the WHO FCTC. These are evidence-based measures that countries can implement to decrease tobacco use and reduce the consequences associated with tobacco use.^30^ WHO FCTC tobacco demand-reduction measures analysed for country investment cases include the following: tax increases; smoke-free indoor air laws; regulations requiring graphical health warnings to be placed on tobacco packaging; regulations regarding plain packaging for tobacco products; bans on tobacco advertising, promotion and sponsorship; and mass media campaigns. For more information about specific measures included in tobacco control investment cases, evolutions in the methodology of policies modelled over time, and the inclusion of custom analyses for certain countries, please refer to the previous paper detailing the methodology for the investment cases^25^ or the investment case reports prepared for each country that are available online.^27^

Specific WHO FCTC tobacco demand-reduction measures were included in a country’s investment case if the country had not yet enacted the measure or if the country had enacted the measure but at a level below WHO FCTC implementation guidelines. For most measures, the impact of each measure on reducing smoking prevalence was obtained from Levy *et al* 31, as adapted by WHO for Appendix 3 of the WHO Global Action Plan for the Prevention and Control of Non-Communicable Diseases 2013–2030.^30–32^ For tax increases, price elasticities of demand were drawn from country and regional elasticity studies or otherwise from a global modelling study that derived estimates.^32 33^ Prevalence elasticities were assumed to be half of price elasticity.^32^ The impact of healthcare providers offering patients brief advice to quit in primary care settings was assessed following methods from Levy*et al*.^34^ The financial costs to government to implement and enforce each of the tobacco demand-reduction measures were estimated using an updated version of the WHO NCD Costing Tool.^35^

The WHO FCTC tobacco demand-reduction measures are intended to work together and complement each other to reduce tobacco use. The measures operate in different ways on different outcomes to change tobacco use behaviours and societal norms regarding tobacco use. In addition to examining the effects of individual tobacco demand-reduction measures on tobacco use, we also examined the combined effects of simultaneously implementing and enforcing multiple tobacco demand-reduction measures to achieve full WHO FCTC implementation. Together, the set of WHO FCTC tobacco demand-reduction policy measures included in the WHO FCTC tobacco control investment case for each country makes up a ‘package’ of WHO FCTC tobacco demand-reduction measures modelled for each country. As noted above, the specific WHO FCTC tobacco demand-reduction measures modelled as part of each country’s WHO FCTC tobacco control investment case were based on what policies and measures countries already had in place at the time the investment case was conducted. As such, the specific WHO FCTC tobacco demand-reduction measures included in each country’s policy package differed among countries. To consider the impact of multiple tobacco demand-reduction measures operating simultaneously, we applied constant proportional reductions following established methods.^26 31^ This means that the combined impact on smoking prevalence from implementing multiple tobacco demand reduction policies as part of the policy package is less than the sum of the effect sizes of the individual measures.^26 31^ Tobacco control programme costs were also included when estimating the costs of simultaneously implementing and enforcing all measures as a package.^26^

The potential benefits of implementing WHO FCTC tobacco demand-reduction measures were evaluated using a counterfactual analysis that forecasted health outcomes (ie, deaths) and socio-economic outcomes (ie, smoking-attributable healthcare expenditures, workplace productivity losses and value of life years lost due to tobacco-attributable mortality) for a 15-year period in the future under two scenarios: a baseline scenario that assumes no changes in tobacco control efforts or smoking rates, and an intervention scenario assuming full implementation and enforcement of the specific WHO FCTC tobacco demand-reduction measures modelled for each country. The baseline scenario remains constant for the entire 15-year period. For the intervention scenario, the starting PAF for tobacco attributable mortality and morbidity, as well as the starting SAF for healthcare expenditures, were the same as the baseline scenario. The PAF and SAF for each year of the intervention scenarios were then adjusted based on the estimated proportional year-over-year changes in estimated adult smoking prevalence resulting from implementation of FCTC tobacco demand-reduction measures.

The modelled effects of tobacco demand reduction policies on smoking prevalence are not immediate. The first 2 years for the intervention scenario are assumed to represent time for planning and implementation, during which time adult smoking prevalence, as well as health and socioeconomic outcomes, remain unchanged. Over the next 3 years, the WHO FCTC tobacco demand-reduction measures begin to affect smoking rates and scale up during a period of initial implementation and enforcement. The last 10 years are characterised by full implementation and enforcement. Using the adjusted PAF and SAF estimates, the estimated tobacco attributable mortality, morbidity and healthcare expenditures were calculated for each year of the intervention scenario. The estimates of workplace productivity losses were also calculated for each year of the intervention scenario based on the estimated smoking prevalence forecasted for each year of the intervention scenario.^25^

We estimate the benefits for each measure analysed, as well as the entire policy package, by comparing the two scenarios to each other and calculating the difference in outcomes between the two scenarios. Benefits include the number of tobacco-related deaths averted and socioeconomic losses averted through 15 years. Costs include the financial cost to country governments to fully implement and enforce the measures. We evaluated the return-on-investment (ROI) for the WHO FCTC tobacco demand-reduction measures by comparing the discounted benefits (eg, the socioeconomic value of tobacco-attributable losses that would be averted over a 15-year period) to the discounted costs (eg, the financial cost to the government over a 15-year period to plan, implement and enforce the measure).^25^

### Analysing and synthesising results across 21 country-specific WHO FCTC tobacco control investment cases

We began our analysis of the results from each of the 21 national WHO FCTC tobacco control investment cases completed between 2017 and 2022 as part of the FCTC 2030 project by aggregating the data inputs and results from each of those investment cases. We grouped the 21 countries by World Bank income group to provide context for the results.^36 37^ Of the 21 countries included in the study, three were low-income countries (LICs) [2020 gross national income (GNI) per capita ≤US$1045], 12 were lower-middle-income countries (LMICs) [2020 GNI per capita between US$1046 and $4095] and 6 were upper-middle-income countries (UMICs) [2020 GNI per capita between US$4096 and $12 695] as of 2022.

Each country-specific tobacco control investment case presents currency values in local currency units. For this synthesis, we adjusted all currency values for inflation using country-specific consumer price index data and then converted all inflation-adjusted local currency values into US$ using data on the official exchange rate (local currency unit per US$). Consumer Price Index and exchange rate data used for these adjustments are from International Monetary Fund data reported through the World Bank’s World Development Indicators database.^38^ All currency values presented in this analysis are expressed in real, inflation adjusted, 2021 US$.

To synthesise findings across the tobacco control investment cases, we first descriptively examine differences across countries related to population characteristics, smoking prevalence, the current health burden of tobacco use and the current socioeconomic costs of tobacco use. To estimate the relationship between adult smoking rates and the socioeconomic burden of tobacco use, we used the data for each of the 21 countries to estimate a simple linear regression model with total annual socioeconomic costs as a percent of GDP as a function of adult smoking prevalence. Finally, we examined the benefits of WHO FCTC tobacco demand-reduction measures and the ROI for those measures by comparing tobacco investment case results across the 21 countries included in our analysis.

## Results

### Descriptive statistics

**Figure 1:**
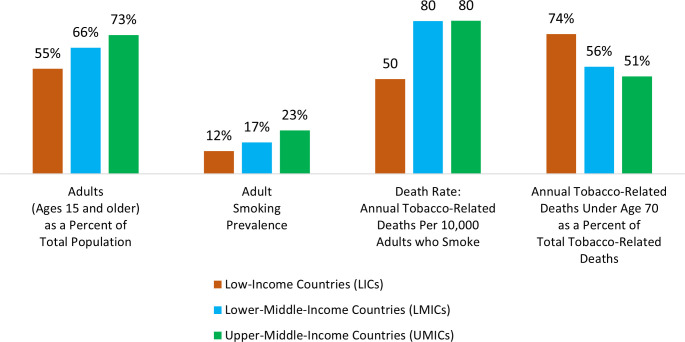
Average adult population, smoking prevalence and smoking-related mortality by country income group.

Included countries vary geographically, demographically and economically. Table 1 presents descriptive statistics for each country and averages by income group. The proportion of adults in the population is generally higher in included LMICs and UMICs compared with included LICs: adults represent an average of 55% of the population among included LICs, 66% among included LMICs and 73% among included UMICs (figure 1).

**Table 1:** Country background: income, population, measures of adult smoking, total healthcare expenditures and employment rate by country

Country	GDP per capita (US$)*	Total population†	Adults (ages 15 and older) as a percent of total population‡	Adult smoking prevalence§	No of adults who smoke¶	Annual tobacco-related deaths per 10 000 adults who smoke**	Total healthcare expenditures per capita (US$)††	Employment rate‡‡
Low-income countries								
Chad	US$900	15 million	50%	5%	406 000	61	US$38	38%
Madagascar	US$500	26 million	57%	15%	2.1 million	39	US$26	85%
Sierra Leone	US$600	7 million	59%	16%	665 000	50	US$108	62%
Average	US$700	16 million	55%	12%	1.1 million	50	US$58	62%
Lower-middle-income countries								
Cabo Verde	US$3200	546 000	72%	9%	36 000	29	US$167	52%
Cambodia	US$1500	16 million	69%	24%	2.5 million	60	US$86	80%
El Salvador	US$4600	6 million	73%	9%	398 000	41	US$307	58%
Eswatini	US$4200	1 million	50%	7%	37 000	178	US$261	41%
Ghana	US$2500	31 million	63%	2%	430 000	156	US$96	63%
Laos	US$2500	7 million	68%	28%	1.3 million	52	US$57	37%
Myanmar	US$1400	55 million	66%	26%	9.4 million	68	US$69	65%
Nepal	US$900	30 million	68%	18%	3.7 million	66	US$50	84%
Samoa	US$4000	201 000	62%	26%	32 000	71	US$241	37%
Sri Lanka	US$3900	21 million	76%	15%	1.7 million	118	US$108	54%
Tunisia	US$3900	12 million	75%	22%	2.0 million	68	US$282	41%
Zambia	US$1200	17 million	55%	14%	1.3 million	57	US$60	33%
Average	US$2800	16 million	66%	17%	1.9 million	80	US$149	54%
Upper-middle-income countries								
Armenia	US$4800	3 million	75%	26%	579 000	95	US$437	47%
Colombia	US$5600	51 million	74%	9%	3.3 million	105	US$316	64%
Costa Rica	US$11 700	5 million	78%	9%	348 000	70	US$865	55%
Georgia	US$3400	4 million	81%	31%	1.0 million	112	US$260	54%
Jordan	US$3900	11 million	55%	43%	2.5 million	36	US$326	42%
Suriname	US$6100	576 000	75%	20%	86 000	64	US$439	48%
Average	US$5900	12 million	73%	23%	1.3 million	80	US$441	52%

All currency values are expressed as real, inflation-adjusted, 2021 US$.

GDP, gross domestic product.

*Calculated using national data sources for GDP and population provided by country partners. In cases when country partners did not provide national data on GDP, country-specific GDP was obtained from the World Bank’s World Development Indicators database.^38^ In cases when country partners did not provide national data on population, country-specific population was obtained from the Global Burden of Disease (GBD) study.^28^ The resulting currency values were adjusted for inflation using country-specific Consumer Price Index data and then all inflation-adjusted local currency values were converted into US$ using data on the official exchange rate (local currency unit per US$). Consumer Price Index and exchange rate data used for these adjustments are from International Monetary Fund data reported through the World Bank’s World Development Indicators database.^38^

†Obtained from national data sources provided by country partners. In cases when country partners did not provide national data on population, country-specific population data were obtained from the GBD study.^28^

‡Calculated using the population data obtained for this study.

§Obtained from national data sources provided by country partners. In cases when country partners did not provide data on smoking prevalence, country-specific adult smoking prevalence was obtained from the country profile from the most recent version of the WHO report on the global tobacco epidemic available at the time when the tobacco control investment case was conducted.

¶Calculated using national data on adult population and adult smoking prevalence obtained for this study.

**Country-specific estimates of annual tobacco-related deaths were obtained from the GBD study.^28^ The rate of tobacco-related deaths per 10 000 adults who smoke was calculated using GBD data on the total number of tobacco-related deaths and the calculated number of adults who smoke.

††Calculated using data on national healthcare expenditures obtained from national data sources provided by country partners. In cases when country partners did not provide national data on healthcare expenditures, country-specific healthcare expenditures were obtained from the WHO Global Health Expenditures Database.^29^ Per capita expenditures were calculated using the population data obtained for this study.

‡‡Obtained from national data sources provided by country partners. In cases when country partners did not provide national data on employment rates, country-specific employment rates were obtained from the World Bank’s World Development Indicators database.^38^

Overall spending on healthcare, including but not limited to spending on treatment and care for tobacco-related illness, varied considerably. Per capita healthcare expenditures averaged US$58 among included LICs, US$149 among included LMICs and US$441 among included UMICs. Average adult smoking prevalence rose for each country income group: 12% among included LICs, 17% among included LMICs and 23% among included UMICs. The absolute number of tobacco-attributable deaths is a function of population size and adult smoking rates. To facilitate comparisons, we present a rate of tobacco-attributable deaths per 10 000 adults who smoke. The average rate of tobacco-related deaths per 10 000 adults who smoke also rose for each country income group: 50 per 10 000 adults who smoke among included LICs and 80 per 10 000 adults who smoke among included LMICs and UMICs. Countries in the higher-income groups have higher smoking-related death rates, as would be expected given higher smoking rates. At the same time, countries in higher-income groups had a lower proportion of smoking-related deaths under age 70, given their older populations and older people who smoke. LICs had a comparatively higher proportion of tobacco-related deaths occurring among individuals under age 70. An average of nearly three-quarters of tobacco-related deaths (74%) among included LICs were comprised of individuals under age 70, in contrast to 56% among included LMICs and 51% among included UMICs.

### Estimated socioeconomic burden of tobacco use

Tobacco use is associated with average socioeconomic losses of US$95 million among included LICs, US$610 million among included LMICs and US$1.6 billion among included UMICs (table 2). These losses amount, respectively, to an average of 1.1%, 1.8% and 2.9% of annual GDP. Annual socioeconomic costs per adult who smokes averaged US$109 among included LICs—ranging from US$73 in Madagascar to US$149 in Chad—US$495 among included LMICs—ranging from US$178 in Nepal to US$1367 in Eswatini—and US$1101 among included UMICs—ranging from US$338 in Georgia to US$1936 in Costa Rica.

**Table 2:** Annual socioeconomic costs of tobacco use

Country	Total annual socioeconomic costs of tobacco use			Direct costs	Indirect costs	
	Total amount (US$)	As a percent of GDP	Amount per adult who smokes (US$)	Smoking-related healthcare expenditures	Social losses: economic cost of tobacco-related mortality	Workplace productivity losses
Low-income countries						
Chad	US$61 million	0.5%	US$149	16%	64%	20%
Madagascar	US$156 million	1.3%	US$73	6%	48%	47%
Sierra Leone	US$69 million	1.5%	US$103	30%	47%	22%
Average	US$95 million	1.1%	US$109	17%	53%	30%
Lower-middle-income countries						
Cabo Verde	US$18 million	1.1%	US$510	8%	27%	66%
Cambodia	US$744 million	3.1%	US$296	10%	42%	48%
El Salvador	US$278 million	1.0%	US$700	44%	35%	21%
Eswatini	US$51 million	1.1%	US$1367	10%	77%	13%
Ghana	US$136 million	0.2%	US$317	26%	52%	22%
Laos	US$418 million	2.4%	US$318	7%	63%	30%
Myanmar	US$2.6 billion	3.4%	US$281	12%	49%	39%
Nepal	US$666 million	2.5%	US$178	8%	40%	52%
Samoa	US$19 million	2.4%	US$592	4%	70%	26%
Sri Lanka	US$1.3 billion	1.6%	US$768	8%	83%	9%
Tunisia	US$818 million	1.8%	US$419	8%	78%	14%
Zambia	US$239 million	1.2%	US$191	7%	50%	43%
Average	US$610 million	1.8%	US$495	13%	55%	32%
Upper-middle-income countries						
Armenia	US$593 million	4.1%	US$1023	28%	57%	15%
Colombia	US$5.2 billion	1.9%	US$1589	39%	50%	11%
Costa Rica	US$674 million	1.1%	US$1936	37%	50%	12%*
Georgia	US$343 million	2.5%	US$338	40%	12%	48%
Jordan	US$2.5 billion	6.0%	US$977	13%	25%	62%
Suriname	US$64 million	1.8%	US$745	10%	67%	22%
Average	US$1.6 billion	2.9%	US$1101	28%	44%	29%

All currency values are expressed as real, inflation adjusted, 2021 US$.

GDP, gross domestic product.

*Smoking breaks were not included in the analysis.

Smoking-related healthcare expenditures accounted for an average of 17% of total tobacco-related costs among included LICs, ranging from 6% in Madagascar to 30% in Sierra Leone. Among included LMICs, smoking-related healthcare expenditures accounted for an average of 13% of total tobacco-related costs, ranging from 4% in Samoa to 44% in El Salvador. Among included UMICs, smoking-related healthcare expenditures accounted for an average of 28% of total tobacco-related costs, ranging from 10% in Suriname to 40% in Georgia. Smoking-attributable healthcare expenditures as a share of total socioeconomic costs associated with tobacco use was less than 30% in 16 of the 21 countries.

The value associated with lives lost due to tobacco use typically accounted for the largest share of losses, averaging at least 50% of the total socioeconomic losses in 12 of the 21 included countries. Mortality costs as a share of the total socioeconomic costs of tobacco use in included LICs ranged from 47% of total costs in Sierra Leone to 64% of total costs in Chad. Among included LMICs, these costs ranged from 27% in Cabo Verde to 83% in Sri Lanka. Among included UMICs, these costs ranged from 12% in Georgia to 67% in Suriname.

Workplace productivity losses associated with tobacco use accounted for an average of 30% of the total burden of tobacco use across the included LICs, ranging from 20% of total costs in Chad to 47% of total costs in Madagascar. Among included LMICs, the average workplace losses associated with tobacco was 32% of the total burden of tobacco use, ranging from 9% in Sri Lanka to 66% in Cabo Verde. Among included UMICs, the average was 29%, ranging from 11% in Colombia to 62% in Jordan. Workplace productivity losses accounted for less than 30% of total costs in 12 of the 21 countries included. Online supplemental table 2 contains more information on the burden of tobacco use by type of loss.

**Figure 2:**
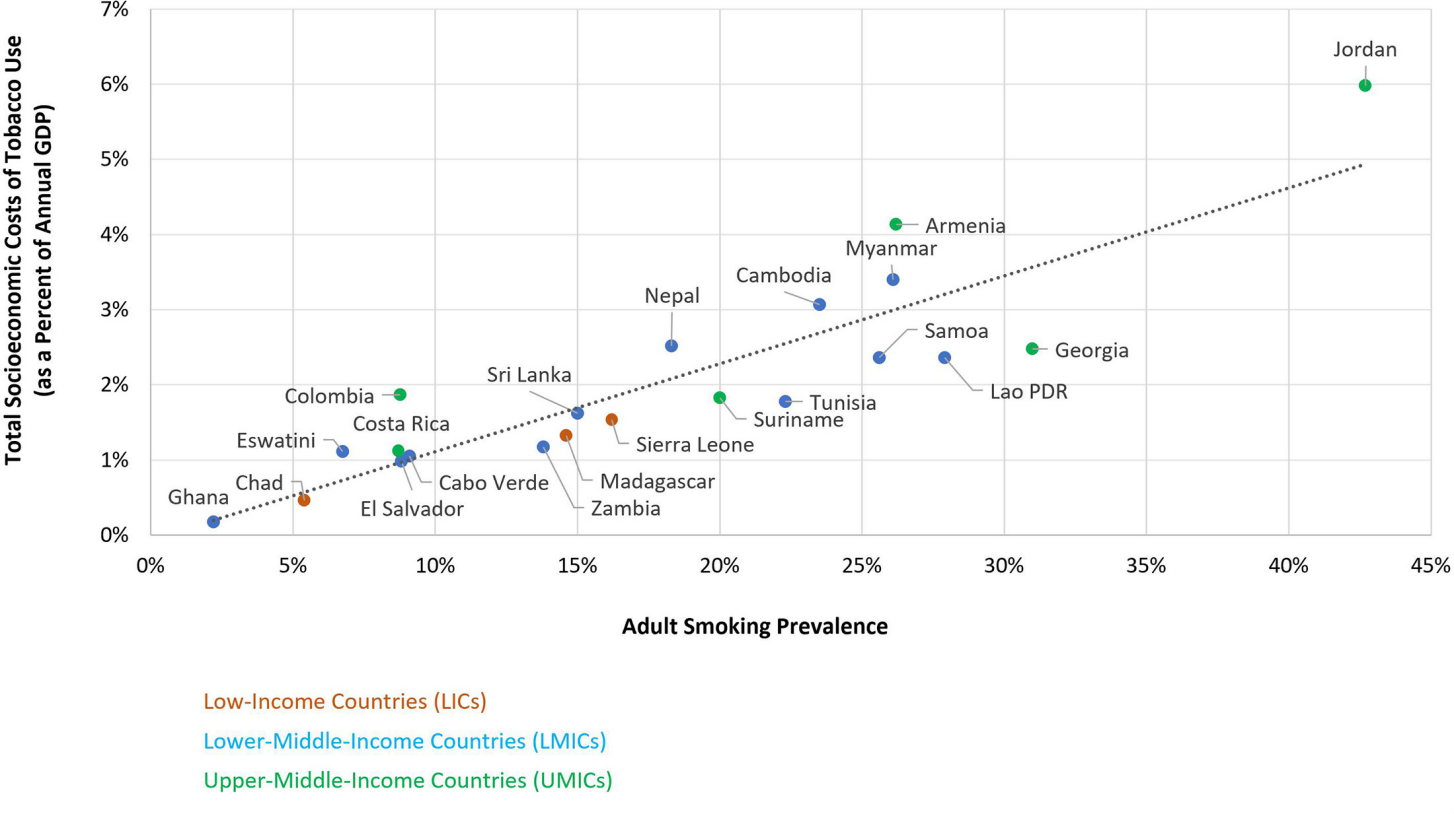
Smoking prevalence and annual socioeconomic costs of tobacco use by country. GDP, gross domestic product.

Figure 2 plots title adult smoking prevalence and total socioeconomic losses associated with tobacco use as a percentage of annual GDP, revealing a clear linear relationship. Increased smoking rates correspond to increased socioeconomic losses as a percentage of GDP. A simple linear regression model confirms this positive relationship between adult smoking prevalence and total socioeconomic losses associated with tobacco use as a percentage of GDP (R^2^=0.79).

### Benefits of WHO FCTC tobacco demand-reduction measures

We assessed the combined effects of implementing a package of WHO FCTC tobacco demand-reduction measures. As noted in the methods section, the policy package included different WHO FCTC tobacco demand-reduction measures based on which measures countries already had in place. Table 3 lists the specific measures that were included in the WHO FCTC tobacco control investment cases and the number of countries that included each measure as part of their WHO FCTC tobacco control investment case.

**Table 3:** WHO FCTC tobacco demand-reduction measures and other policy actions modelled in WHO FCTC tobacco control investment cases

Measure/policy action	Total	LICs	LMICs	UMICs
Total no of countries	21	3	12	6
WHO FCTC tobacco demand-reduction measures				
Raise cigarette taxes (FCTC Art. 6)	21	3	12	6
Protect people from tobacco smoke (FCTC Art. 8)*	21	3	12	6
Warning labels (FCTC Art. 11)	11	1	6	4
Plain packaging (FCTC Art. 11 guidelines/Art. 11)	20	3	12	5
Bans on advertising (FCTC Art. 11/13)*	16	1	11	4
Mass media campaigns (FCTC Art. 12)	15	3	9	3
Cessation support: brief advice to quit tobacco (FCTC Art. 14)†	8	0	6	2
Additional policy actions modelled for specific countries‡				
Cessation support: establish a toll-free national tobacco quitline	1	0	1	0
Ban single stick cigarettes	1	0	1	0

*Some countries had implemented these measures at levels recommended under WHO FCTC guidelines, but without adequate enforcement. In these cases, the investment case analyses assessed the impact of improved enforcement.

†Initially added as a custom intervention for a specific country investment case after several other tobacco control investment cases were already completed. This measure was subsequently made available to other countries and became a routine component of tobacco control investment cases later in the process of completing investment cases.

‡Additional measures analysed for specific countries as part of their FCTC tobacco control investment case.

LICs, low-income countries; LMICs, lower-middle-income countries; UMICs, upper-middle-income countries; WHO FCTC, WHO Framework Convention on Tobacco Control.

Table 4 presents the number of tobacco demand-reduction measures included in the policy package for each country along with adult smoking prevalence for each country at the start and end of the modelled 15-year period. The table also presents the relative reduction in adult smoking prevalence and the total number of tobacco-related deaths and socioeconomic losses averted with full implementation and enforcement of the modelled WHO FCTC measures.

**Table 4:** Impact of FCTC tobacco demand-reduction policy package over 15 years

Country	Measures in policy package	Adult smoking prevalence over 15 years			Tobacco-related deaths averted over 15 years (with package)	Socioeconomic losses averted (US$) over 15 years (with package)	Financial cost of implementing package (US$) over 15 years	Return on investment
		Starting	Ending	Relative change				
Low-income countries								
Chad	4	5%	3%	−47%	11 000	US$198 million	US$15 million	13
Madagascar	4	15%	8%	−42%	30 000	US$443 million	US$17 million	25
Sierra Leone	6	16%	5%	−69%	20 000	US$315 million	US$12 million	27
Average	5	12%	5%	−53%	20 000	US$319 million	US$15 million	22
Lower-middle-income countries								
Cabo Verde	6	9%	3%	−63%	600	US$78 million	US$10 million	8
Cambodia	5	24%	13%	−43%	57 000	US$2.2 billion	US$12 million	181
El Salvador	7	9%	5%	−48%	7000	US$886 million	US$21 million	43
Eswatini	7	7%	3%	−59%	3000	US$201 million	US$13 million	15
Ghana	5	2%	1%	−33%	20 000	US$269 million	US$61 million	4
Laos	7	28%	14%	−49%	25 000	US$1.2 billion	US$14 million	86
Myanmar	5	26%	14%	−45%	255 000	US$8.1 billion	US$35 million	233
Nepal	4	18%	11%	−39%	84 000	US$1.7 billion	US$24 million	71
Samoa	5	26%	14%	−46%	1000	US$61 million	US$6 million	10
Sri Lanka	6	15%	9%	−40%	72 000	US$3.6 billion	US$21 million	172
Tunisia	7	22%	12%	−46%	56 000	US$2.3 billion	US$36 million	63
Zambia	6	14%	5%	−64%	40 000	US$1.0 billion	US$25 million	42
Average	6	17%	9%	−48%	52 000	US$1.8 billion	US$23 million	78
Upper-middle-income countries								
Armenia	7	26%	13%	−52%	23 000	US$1.9 billion	US$23 million	85
Colombia	4	9%	4%	−50%	154 000	US$17.9 billion	US$58 million	310
Costa Rica	4	9%	6%	−35%	8000	US$1.4 billion	US$12 million	118
Georgia	4	31%	17%	−45%	53 000	US$1.5 billion	US$4 million	364
Jordan	6	43%	18%	−57%	48 000	US$10.1 billion	US$41 million	247
Suriname	5	20%	13%	−36%	2000	US$137 million	US$10 million	14
Average	5	23%	12%	−46%	48 000	US$5.5 billion	US$25 million	190

All currency values are expressed as real, inflation adjusted, 2021 US$.

The WHO FCTC tobacco demand-reduction measures are estimated to result in fewer tobacco-related deaths, with an average of 20 000 averted deaths over a 15-year period among included LICs, 52 000 among included LMICs and 48 000 among included UMICs—varying based on population size, smoking rates and the core package of measures that were modelled for each country. With complete implementation of the WHO FCTC measures, an average of US$319 million in socioeconomic losses could be averted among included LICs, US$1.8 billion among included LMICs and US$5.5 billion among included UMICs. The ROI for the WHO FCTC tobacco demand-reduction policy package is positive for every one of the 21 countries. Socioeconomic losses averted through the full implementation and enforcement of the tobacco control policy package would be considerably greater than the financial costs associated with implementing and enforcing the package. Policy-specific results for each modelled demand-reduction measure included in each country’s package are available in online supplemental table 3.

## Discussion

This study describes and compares the results of 21 country-specific investment cases for tobacco control to identify common themes across countries. In all studied cases, the benefits of investments in tobacco control greatly outweigh the costs of implementation. Our results confirm a strong correlation between smoking prevalence and cost. Countries with the highest smoking prevalence experience the greatest socioeconomic losses from tobacco use. Irrespective of the varied contexts, country customisations and methodological evolution, tobacco is harmful to the health and economy of all countries included in this analysis.

The 21 included countries are diverse, representing various geographies, population sizes and country income levels. We document several patterns related to tobacco consumption. First, adult smoking prevalence is higher in wealthier country income groups included in our analysis. The average adult smoking prevalence in UMICs is almost double the prevalence in LICs (23% and 12%, respectively). This suggests that as economies and incomes grow, individuals use some of their additional disposable income to purchase cigarettes. This highlights the importance of early implementation of tobacco control measures in LICs. Preventing increases in smoking prevalence can reduce socioeconomic losses, which this analysis shows grow (ie, costs per adult who smokes increase) as country incomes increase.

Second, tobacco-related deaths among adults who smoke also correlate with smoking rates. LMICs and UMICs, which had higher smoking prevalence, also had higher rates of tobacco-related deaths than LICs. Even so, the relative share of socioeconomic losses due to mortality decreased as country incomes increased, with tobacco-attributable healthcare expenditures rising to take its place. As health systems strengthen alongside rising country incomes and specialised care services become more accessible, tobacco use will increase health system use and expenditures. For overburdened health systems that may not have capacity to treat rising cases of complex diseases, and governments and families facing catastrophic health expenditures, averting preventable disease cases by reducing tobacco use is of paramount importance.

Third, the age at which tobacco-related deaths occur correlates with country income. A higher proportion of tobacco-related deaths in LMICs and UMICs (56% and 51%, respectively) occurred among older age groups, compared with LICs (74%). In countries with less-developed health systems, people may not readily receive preventative or curative care for infectious or non-communicable diseases. This compounds the burden caused by tobacco use. In all countries, but especially those with low life expectancy, decreasing tobacco use can contribute to preventing deaths under age 70 while also stimulating economic growth.^39^

The ROI for the WHO FCTC tobacco demand-reduction policy package was greater than 1 for every one of the 21 countries in this analysis. The ROI was also greater than 1 for almost every single policy analysed (as shown in online supplemental table 3). All 21 tobacco control investment cases included an increase in taxes and protecting people from tobacco smoke, 20 included plain packaging, 16 included bans on advertising, 15 included mass media campaigns, 11 included graphic warning labels and 8 included brief advice to quit in primary care settings. One country’s investment case included an assessment of implementing a national tobacco cessation hotline, and another country’s investment case included an assessment of banning single stick cigarette sales. Increasing taxation on tobacco products was consistently the policy with the highest ROI due to its effectiveness in reducing prevalence and low cost of implementation. While there are valid concerns with tax policies, those issues often revolve around the way tax increases are implemented. Some critiques involve the potential for tax increases on tobacco products to be regressive and impose a greater financial burden on tobacco users with lower income. As discussed further in another paper in this collection, taxation can lead to increased equity, as people with lower-income who smoke quit at higher rates in response to increases in price compared with people with higher-income who smoke—meaning that those same people with lower-income who smoke receive a disproportionate benefit of the health and economic benefits of smoking cessation.^40^ This is particularly relevant when governments use proceeds from tobacco tax revenues to fund effective tobacco control programmes that directly provide evidence-based services and programmes that help those with lower-income successfully quit using tobacco.^41 42^

The ROI for a policy is a combination of both effectiveness and cost. Policies that have higher effectiveness and lower cost will have the highest ROI. A lower ROI could be due to higher policy implementation and enforcement costs relative to other policies, even if the policy is equally as effective at reducing tobacco use. For example, the estimated reductions in smoking prevalence associated with bans on tobacco advertising, promotion and sponsorship tended to be similar to the impacts of running mass media campaigns. However, the mass media campaigns tended to have lower ROI because they were associated with considerably higher costs for implementation.

We would caution that our results should not be interpreted to suggest that interventions with higher ROIs are the only measures worth pursuing. Countries that have successfully achieved sustained reductions in tobacco prevalence have not done so through one measure alone. Tobacco control measures are intended to work together and complement each other to reduce tobacco use. While specific measures may not produce the largest reductions in tobacco use, they are still an important part of larger comprehensive tobacco control efforts. Ultimately, the best policies are the ones that countries can pass, implement and enforce. Relevant background information and evidence for the proven WHO FCTC demand-reduction measures can be found in the 2022 update to Appendix 3 of the WHO Global NCD Action Plan 2013–2030.^43^

This analysis provides a comparison of tobacco control investment cases from 21 diverse countries. It is not without limitations. One of the biggest challenges for conducting national tobacco control investment cases is finding and obtaining the data required to conduct the analysis. When possible, national data inputs were obtained directly from government stakeholders and country partners working on the investment cases. In many cases, national data were not available directly from country partners but were available from global data sources that reported country-specific data. Examples include the Global Burden of Disease Study or various WHO and World Bank data. Sometimes, national data were not available from any source, in which case regional or even global averages were used. Data frequency was also an issue. Some of the data inputs and parameter values used for the tobacco control investment cases were dated—demonstrating the continued need for funding and implementing regular surveillance. In addition, the starting point and combination of interventions were different for each country, with some countries starting nearly from scratch and others modelling the impact of increased coverage or enforcement of policies that were already in place. These limitations create some issues when comparing results across countries. Countries that had already implemented many tobacco control measures, and/or that have comparatively low smoking prevalence, still had positive returns associated with further investments in tobacco control.

Despite limitations, the results from the 21 tobacco control investment cases completed between 2017 and 2022 are qualitatively similar and tell a consistent story. Each national tobacco control investment case clearly demonstrates the tremendous health and socioeconomic burden that tobacco use places on countries and establishes clear motivation to take action to reduce tobacco use. While the magnitude of the positive returns on investment in effective tobacco control measures may vary, in all 21 countries in this analysis the costs of fully implementing and enforcing proven, evidence-based WHO FCTC tobacco control policies are greatly outweighed by the health and socioeconomic benefits that could be achieved as a result of fully implementing and enforcing those policies.

## Data Availability

Data sharing not applicable as no datasets generated and/or analysed for this study.
