# Supplementary material for: Interpreting results, impacts and implications from WHO FCTC tobacco control investment cases in 21 low-income and middle-income countries
Source: Tob Control. 2024 May 2;33(Suppl 1):s17–26. doi: 10.1136/tc-2023-058337 (PMC11103323; doi:10.1136/tc-2023-058337)
Supplement: Supplementary data [file tc-2023-058337supp001.pdf]

# Interpreting results, impacts and implications from WHO FCTC tobacco control investment cases in 21 low-income and middle-income countries

## Supplementary Materials

## Supplemental Table 1. Model Inputs and Parameters for Estimating the Annual Burden of Tobacco Use

| | Tobacco Use, Tobacco-Related Mortality, and the Value of Lost Life | | | | | Smoking-Related Healthcare Expenditures | | | Smoking-Related Workplace Productivity Losses | | | | |
|---|---|---|---|---|---|---|---|---|---|---|---|---|---|
| Country | Adult smoking prevalence | Annual Tobacco -Related Deaths | Annual Tobacco-Related Deaths Per 10,000 Adults who Smoke | Percent of Annual Tobacco-Related Deaths Under Age 70 | Social Value of a Life Year (US$) | Total Healthcare Expenditures (US$) | Total Healthcare Expenditures Per Capita (US$) | Smoking-Attributable Fraction (SAF) of Healthcare Expenditures | Employment Rate | Average Annual Wages (US$) | Excess Absenteeism (Days) | Excess Presenteeism (Percent of Working Time) | Unsanctioned Smoking Breaks (Minutes Per Day) |
| **Low-Income Countries** | | | | | | | | | | | | | |
| Chad | 5% | 2,500 | 61 | 71% | $1,200 | $577 million | $38 | 1.7% | 38% | $1,300 | 2.6 | 3.1% | 10 |
| Madagascar | 15% | 8,300 | 39 | 82% | $600 | $684 million | $26 | 1.3% | 85% | $600 | 2.6 | 3.1% | 10 |
| Sierra Leone | 16% | 3,300 | 50 | 68% | $900 | $769 million | $108 | 2.7% | 62% | $600 | 2.6 | 3.1% | 10 |
| **Average** | **12%** | **4,700** | **50** | **74%** | **$900** | **$0.7 billion** | **$58** | **1.9%** | **62%** | **$800** | **2.6** | **3.1%** | **10** |
| **Lower-Middle-Income Countries** | | | | | | | | | | | | | |
| Cabo Verde | 9% | 100 | 29 | 60% | $4,400 | $91 million | $167 | 1.5% | 52% | $9,000 | 2.6 | 3.1% | 10 |
| Cambodia | 24% | 15,100 | 60 | 55% | $2,100 | $1.4 billion | $86 | 5.4% | 80% | $2,500 | 3.0 | 3.1% | 10 |
| El Salvador | 9% | 1,600 | 41 | 46% | $6,400 | $1.9 billion | $307 | 6.5% | 58% | $3,100 | 3.0 | 3.1% | 10 |
| Eswatini | 7% | 700 | 178 | 66% | $5,800 | $286 million | $261 | 1.7% | 41% | $4,400 | 2.9 | 3.1% | 10 |
| Ghana | 2% | 6,700 | 156 | 66% | $1,100 | $3.0 billion | $96 | 1.2% | 63% | $2,000 | 2.9 | 3.1% | Not Included |
| Lao PDR | 28% | 6,800 | 52 | 61% | $3,600 | $398 million | $57 | 7.0% | 37% | $3,400 | 2.9 | 3.1% | 10 |
| Myanmar | 26% | 64,000 | 68 | 56% | $2,000 | $3.8 billion | $69 | 8.5% | 65% | $2,300 | 2.6 | 3.1% | 10 |
| Nepal | 18% | 24,800 | 66 | 47% | $1,200 | $1.5 billion | $50 | 3.3% | 84% | $1,800 | 2.6 | 3.1% | 10 |
| Samoa | 26% | 200 | 71 | 56% | $5,600 | $48 million | $241 | 1.5% | 37% | $6,400 | 2.6 | 3.1% | 10 |
| Sri Lanka | 15% | 20,000 | 118 | 55% | $5,400 | $2.2 billion | $108 | 4.6% | 54% | $1,800 | 3.0 | 3.1% | 5 |
| Tunisia | 22% | 13,300 | 68 | 48% | $5,500 | $3.3 billion | $282 | 1.9% | 41% | $1,600 | 2.9 | 3.1% | 10 |
| Zambia | 14% | 7,100 | 57 | 60% | $1,700 | $1.0 billion | $60 | 1.6% | 33% | $3,300 | 2.6 | 3.1% | 10 |
| **Average** | **17%** | **13,400** | **80** | **56%** | **$3,733** | **$1.6 billion** | **$149** | **3.7%** | **54%** | **$3,500** | **2.8** | **3.1%** | **10** |
| **Upper-Middle-Income Countries** | | | | | | | | | | | | | |
| Armenia | 26% | 5,500 | 95 | 52% | $6,800 | $1.3 billion | $437 | 12.6% | 47% | $4,700 | 2.9 | 3.1% | 10 |
| Colombia | 9% | 34,800 | 105 | 44% | $7,800 | $16.0 billion | $316 | 12.8% | 64% | $4,300 | 2.6 | 3.1% | 8 |
| Costa Rica | 9% | 2,400 | 70 | 41% | $16,400 | $4.4 billion | $865 | 5.7% | 55% | $9,100 | 2.9 | 3.1% | Not Included |
| Georgia | 31% | 11,400 | 112 | 52% | $4,800 | $1.0 billion | $260 | 13.0% | 54% | $4,400 | 2.6 | 1.0% | 15 |
| Jordan | 43% | 9,000 | 36 | 58% | $5,400 | $3.5 billion | $326 | 9.1% | 42% | $8,800 | 4.0 | 4.0% | 16 |
| Suriname | 20% | 500 | 64 | 62% | $8,500 | $253 million | $439 | 2.6% | 48% | $4,400 | 2.9 | 3.1% | 10 |
| **Average** | **23%** | **10,600** | **80** | **51%** | **$8,283** | **$4.4 billion** | **$441** | **9.3%** | **52%** | **$6,000** | **3.0** | **2.9%** | **12** |

All currency values are expressed as real, inflation-adjusted, 2021 US$.

## Supplemental Table 2. Burden of Tobacco Use: Annual Losses by Type of Loss

| Country | Total Annual Socioeconomic Costs | Direct Costs | | Indirect Costs | | | | | | | |
|---|---|---|---|---|---|---|---|---|---|---|---|
| | | | | Social Losses | | Smoking-Related Workplace Productivity Losses | | | | | |
| | | Smoking-Related Healthcare Expenditures | | Social Cost of Tobacco-Related Mortality | | Absenteeism | | Presenteeism | | Unsanctioned Smoking Breaks | |
| | Value (US$) | Value (US$) | Percent of Total | Value (US$) | Percent of Total | Value (US$) | Percent of Total | Value (US$) | Percent of Total | Value (US$) | Percent of Total |
| **Low-Income Countries** | | | | | | | | | | | |
| Chad | $61 million | $10 million | 16% | $39 million | 64% | $2 million | 3% | $6 million | 10% | $4 million | 7% |
| Madagascar | $156 million | $9 million | 6% | $74 million | 48% | $12 million | 8% | $36 million | 23% | $25 million | 16% |
| Sierra Leone | $69 million | $21 million | 30% | $33 million | 47% | $2 million | 4% | $7 million | 11% | $5 million | 8% |
| **Average** | **$95 million** | **$13 million** | **17%** | **$49 million** | **53%** | **$5 million** | **5%** | **$17 million** | **15%** | **$12 million** | **10%** |
| **Lower-Middle-Income Countries** | | | | | | | | | | | |
| Cabo Verde | $18 million | $1 million | 8% | $5 million | 27% | $2 million | 11% | $6 million | 32% | $4 million | 23% |
| Cambodia | $744 million | $75 million | 10% | $310 million | 42% | $67 million | 9% | $175 million | 24% | $117 million | 16% |
| El Salvador | $278 million | $123 million | 44% | $96 million | 35% | $11 million | 4% | $29 million | 10% | $20 million | 7% |
| Eswatini | $51 million | $5 million | 10% | $39 million | 77% | $1 million | 2% | $3 million | 6% | $2 million | 4% |
| Ghana | $136 million | $36 million | 26% | $70 million | 52% | $8 million | 6% | $22 million | 16% | Not Included | -- |
| Lao PDR | $418 million | $28 million | 7% | $264 million | 63% | $23 million | 5% | $61 million | 15% | $43 million | 10% |
| Myanmar | $2.6 billion | $320 million | 12% | $1.3 billion | 49% | $169 million | 6% | $508 million | 19% | $360 million | 14% |
| Nepal | $666 million | $54 million | 8% | $269 million | 40% | $56 million | 8% | $168 million | 25% | $119 million | 18% |
| Samoa | $19 million | $730,000 | 4% | $13 million | 70% | $900,000 | 5% | $3 million | 14% | $2 million | 8% |
| Sri Lanka | $1.3 billion | $103 million | 8% | $1.1 billion | 83% | $26 million | 2% | $67 million | 5% | $24 million | 2% |
| Tunisia | $818 million | $62 million | 8% | $640 million | 78% | $21 million | 3% | $56 million | 7% | $39 million | 5% |
| Zambia | $239 million | $16 million | 7% | $119 million | 50% | $17 million | 7% | $51 million | 21% | $36 million | 15% |
| **Average** | **$610 million** | **$69 million** | **13%** | **$349 million** | **55%** | **$33 million** | **6%** | **$96 million** | **16%** | **$70 million** | **11%** |
| **Upper-Middle-Income Countries** | | | | | | | | | | | |
| Armenia | $593 million | $163 million | 28% | $339 million | 57% | $16 million | 3% | $44 million | 7% | $31 million | 5% |
| Colombia | $5.2 billion | $2.0 billion | 39% | $2.6 billion | 50% | $103 million | 2% | $310 million | 6% | $176 million | 3% |
| Costa Rica | $674 million | $252 million | 37% | $340 million | 50% | $22 million | 3% | $60 million | 9% | Not Included | -- |
| Georgia | $343 million | $136 million | 40% | $42 million | 12% | $32 million | 9% | $31 million | 9% | $101 million | 30% |
| Jordan | $2.5 billion | $316 million | 13% | $617 million | 25% | $205 million | 8% | $540 million | 22% | $785 million | 32% |
| Suriname | $64 million | $7 million | 10% | $43 million | 67% | $2.5 million | 4% | $7 million | 11% | $5 million | 8% |
| **Average** | **$1.6 billion** | **$487 million** | **28%** | **$666 million** | **44%** | **$63 million** | **5%** | **$165 million** | **11%** | **$220 million** | **16%** |

All currency values are expressed as real, inflation-adjusted, 2021 US$.

## Supplemental Table 3. Impacts of FCTC Tobacco Control Measures in FCTC Tobacco Control Investment Cases by Country

| FCTC Tobacco Control Measure | Included in FCTC Tobacco Control Investment Case | Relative Change in Adult Smoking Prevalence Over 15 Years * | Tobacco-Related Deaths Averted Over 15 Years | Socioeconomic Losses Averted (US$) Over 15 Years | Implementation Costs for Policy/Intervention (US$) Over 15 Years | Return On Investment (ROI) for Policy/Intervention Over 15 Years |
|---|---|---|---|---|---|---|
| **Low-Income Countries** | | | | | | |
| **Chad** | **4 measures** | **-47%** | **10,500** | **$198 million** | **$15.0 million** | **13** |
| Raise cigarette taxes (FCTC Art. 6) | Yes | -27% | 6,100 | $115 million | $2.2 million | 52 |
| Protect people from tobacco smoke (FCTC Art. 8) | Yes | -10% | 2,500 | $48 million | $4.3 million | 11 |
| Warning labels (FCTC Art. 11) | No † | | | | | |
| Plain packaging (FCTC Art. 11 Guidelines / Art. 11) | Yes | -4% | 1,100 | $20 million | $2.1 million | 10 |
| Bans on advertising (FCTC Art. 11/13) | No ‡ | | | | | |
| Mass media campaigns (FCTC Art. 12) | Yes | -16% | 3,900 | $74 million | $3.3 million | 22 |
| Cessation support: brief advice to quit tobacco (FCTC Art. 14) | No | | | | | |
| **Madagascar** | **4 measures** | **-42%** | **30,400** | **$443 million** | **$17.4 million** | **25** |
| Raise cigarette taxes (FCTC Art. 6) | Yes | -25% | 17,200 | $249 million | $2.1 million | 121 |
| Protect people from tobacco smoke (FCTC Art. 8) | Yes | -7% | 6,200 | $90 million | $4.6 million | 19 |
| Warning labels (FCTC Art. 11) | No † | | | | | |
| Plain packaging (FCTC Art. 11 Guidelines / Art. 11) | Yes | -4% | 3,100 | $46 million | $2.2 million | 20 |
| Bans on advertising (FCTC Art. 11/13) | No ‡ | | | | | |
| Mass media campaigns (FCTC Art. 12) | Yes | -14% | 11,500 | $168 million | $5.2 million | 32 |
| Cessation support: brief advice to quit tobacco (FCTC Art. 14) | No | | | | | |
| **Sierra Leone** | **6 measures** | **-69%** | **19,800** | **$315 million** | **$11.7 million** | **27** |
| Raise cigarette taxes (FCTC Art. 6) | Yes | -35% | 10,500 | $165 million | $1.5 million | 113 |
| Protect people from tobacco smoke (FCTC Art. 8) | Yes | -11% | 3,800 | $59 million | $2.4 million | 25 |
| Warning labels (FCTC Art. 11) | Yes | -15% | 5,000 | $78 million | $1.2 million | 65 |
| Plain packaging (FCTC Art. 11 Guidelines / Art. 11) | Yes | -4% | 1,300 | $20 million | $1.2 million | 17 |
| Bans on advertising (FCTC Art. 11/13) | Yes | -29% | 9,500 | $149 million | $1.2 million | 121 |
| Mass media campaigns (FCTC Art. 12) | Yes | -6% | 2,200 | $34 million | $2.5 million | 14 |
| Cessation support: brief advice to quit tobacco (FCTC Art. 14) | No | | | | | |

| FCTC Tobacco Control Measure | Included in FCTC Tobacco Control Investment Case | Relative Change in Adult Smoking Prevalence Over 15 Years * | Tobacco-Related Deaths Averted Over 15 Years | Socioeconomic Losses Averted (US$) Over 15 Years | Implementation Costs for Policy/Intervention (US$) Over 15 Years | Return On Investment (ROI) for Policy/Intervention Over 15 Years |
|---|---|---|---|---|---|---|
| **Lower-Middle-Income Countries** | | | | | | |
| **Cabo Verde** | **6 measures** | **-63%** | **570** | **$78 million** | **$9.5 million** | **8** |
| Raise cigarette taxes (FCTC Art. 6) | Yes | -24% | 200 | $29 million | $1.8 million | 16 |
| Protect people from tobacco smoke (FCTC Art. 8) | Yes | -16% | 200 | $23 million | $1.6 million | 15 |
| Warning labels (FCTC Art. 11) | Yes | -17% | 200 | $24 million | $900,000 | 26 |
| Plain packaging (FCTC Art. 11 Guidelines / Art. 11) | Yes | -6% | 100 | $9 million | $900,000 | 10 |
| Bans on advertising (FCTC Art. 11/13) | Yes | -12% | 100 | $18 million | $950,000 | 19 |
| Mass media campaigns (FCTC Art. 12) | Yes | -16% | 200 | $22 million | $2.3 million | 10 |
| Cessation support: brief advice to quit tobacco (FCTC Art. 14) | No | | | | | |
| **Cambodia** | **5 measures** | **-43%** | **57,200** | **$2.2 billion** | **$12.0 million** | **181** |
| Raise cigarette taxes (FCTC Art. 6) | Yes | -23% | 30,200 | $1.1 billion | $1.3 million | 897 |
| Protect people from tobacco smoke (FCTC Art. 8) | Yes | -6% | 10,100 | $383 million | $2.8 million | 139 |
| Warning labels (FCTC Art. 11) | No † | | | | | |
| Plain packaging (FCTC Art. 11 Guidelines / Art. 11) | Yes | -5% | 7,600 | $289 million | $1.3 million | 221 |
| Bans on advertising (FCTC Art. 11/13) | Yes | -6% | 10,100 | $383 million | $1.3 million | 289 |
| Mass media campaigns (FCTC Art. 12) | Yes | -10% | 16,000 | $607 million | $3.5 million | 176 |
| Cessation support: brief advice to quit tobacco (FCTC Art. 14) | No | | | | | |
| **El Salvador** | **7 measures** | **-48%** | **6,700** | **$886 million** | **$20.5 million** | **43** |
| Raise cigarette taxes (FCTC Art. 6) | Yes | -15% | 1,900 | $248 million | $1.9 million | 127 |
| Protect people from tobacco smoke (FCTC Art. 8) | Yes | -10% | 1,700 | $218 million | $3.1 million | 71 |
| Warning labels (FCTC Art. 11) | Yes | -11% | 1,800 | $233 million | $1.6 million | 147 |
| Plain packaging (FCTC Art. 11 Guidelines / Art. 11) | Yes | -9% | 1,600 | $204 million | $1.6 million | 127 |
| Bans on advertising (FCTC Art. 11/13) | Yes | -11% | 1,800 | $232 million | $1.6 million | 144 |
| Mass media campaigns (FCTC Art. 12) | No † | | | | | |
| Cessation support: brief advice to quit tobacco (FCTC Art. 14) | Yes | -4% | 500 | $70 million | $3.3 million | 21 |
| Cessation support: establish a free national hotline [2] | Yes | -0.2% | 20 | $3 million | $7.4 million | 0.4 |

| FCTC Tobacco Control Measure | Included in FCTC Tobacco Control Investment Case | Relative Change in Adult Smoking Prevalence Over 15 Years * | Tobacco-Related Deaths Averted Over 15 Years | Socioeconomic Losses Averted (US$) Over 15 Years | Implementation Costs for Policy/Intervention (US$) Over 15 Years | Return On Investment (ROI) for Policy/Intervention Over 15 Years |
|---|---|---|---|---|---|---|
| **Eswatini** | **7 measures** | **-59%** | **3,400** | **$201 million** | **$13.3 million** | **15** |
| Raise cigarette taxes (FCTC Art. 6) | Yes | -21% | 1,100 | $67 million | $1.9 million | 36 |
| Protect people from tobacco smoke (FCTC Art. 8) | Yes | -17% | 1,100 | $66 million | $1.7 million | 39 |
| Warning labels (FCTC Art. 11) | Yes | -12% | 800 | $50 million | $970,000 | 52 |
| Plain packaging (FCTC Art. 11 Guidelines / Art. 11) | Yes | -4% | 300 | $17 million | $970,000 | 18 |
| Bans on advertising (FCTC Art. 11/13) | Yes | -8% | 600 | $34 million | $1.0 million | 33 |
| Mass media campaigns (FCTC Art. 12) | Yes | -16% | 1,100 | $63 million | $2.0 million | 32 |
| Cessation support: brief advice to quit tobacco (FCTC Art. 14) | Yes | -3% | 100 | $6 million | $3.7 million | 2 |
| **Ghana** | **5 measures** | **-33%** | **20,100** | **$269 million** | **$60.6 million** | **4** |
| Raise cigarette taxes (FCTC Art. 6) | Yes | -18% | 11,200 | $149 million | $3.5 million | 42 |
| Protect people from tobacco smoke (FCTC Art. 8) | Yes | -6% | 4,600 | $62 million | $10.5 million | 6 |
| Warning labels (FCTC Art. 11) | No | | | | | |
| Plain packaging (FCTC Art. 11 Guidelines / Art. 11) | Yes | -4% | 2,900 | $39 million | $5.1 million | 8 |
| Bans on advertising (FCTC Art. 11/13) | Yes | -6% | 4,300 | $58 million | $5.1 million | 11 |
| Mass media campaigns (FCTC Art. 12) | No | | | | | |
| Cessation support: brief advice to quit tobacco (FCTC Art. 14) | Yes | -3% | 1,100 | $13 million | $28.6 million | 0.5 |
| **Lao PDR** | **7 measures** | **-49%** | **25,300** | **$1.2 billion** | **$13.8 million** | **86** |
| Raise cigarette taxes (FCTC Art. 6) | Yes | -31% | 14,100 | $638 million | $840,000 | 763 |
| Protect people from tobacco smoke (FCTC Art. 8) | Yes | -4% | 3,100 | $145 million | $1.7 million | 84 |
| Warning labels (FCTC Art. 11) | Yes | -2% | 1,600 | $74 million | $880,000 | 84 |
| Plain packaging (FCTC Art. 11 Guidelines / Art. 11) | Yes | -3% | 2,300 | $109 million | $880,000 | 124 |
| Bans on advertising (FCTC Art. 11/13) | Yes | -9% | 6,000 | $286 million | $880,000 | 326 |
| Mass media campaigns (FCTC Art. 12) | Yes | -8% | 5,700 | $272 million | $2.2 million | 125 |
| Cessation support: brief advice to quit tobacco (FCTC Art. 14) | Yes | -3% | 1,300 | $57 million | $5.2 million | 11 |

| FCTC Tobacco Control Measure | Included in FCTC Tobacco Control Investment Case | Relative Change in Adult Smoking Prevalence Over 15 Years * | Tobacco-Related Deaths Averted Over 15 Years | Socioeconomic Losses Averted (US$) Over 15 Years | Implementation Costs for Policy/Intervention (US$) Over 15 Years | Return On Investment (ROI) for Policy/Intervention Over 15 Years |
|---|---|---|---|---|---|---|
| **Myanmar** | **5 measures** | **-45%** | **255,400** | **$8.1 billion** | **$34.7 million** | **233** |
| Raise cigarette taxes (FCTC Art. 6) | Yes | -15% | 79,900 | $2.5 billion | $2.3 million | 1,094 |
| Protect people from tobacco smoke (FCTC Art. 8) | Yes | -14% | 89,200 | $2.8 billion | $7.9 million | 356 |
| Warning labels (FCTC Art. 11) | No † | | | | | |
| Plain packaging (FCTC Art. 11 Guidelines / Art. 11) | Yes | -6% | 36,800 | $1.2 billion | $3.5 million | 331 |
| Bans on advertising (FCTC Art. 11/13) | Yes | -7% | 48,600 | $1.5 billion | $2.2 million | 692 |
| Mass media campaigns (FCTC Art. 12) | Yes | -14% | 90,400 | $2.9 billion | $13.4 million | 213 |
| Cessation support: brief advice to quit tobacco (FCTC Art. 14) | No | | | | | |
| **Nepal** | **4 measures** | **-39%** | **83,900** | **$1.7 billion** | **$24.0 million** | **71** |
| Raise cigarette taxes (FCTC Art. 6) | Yes | -32% | 68,400 | $1.4 billion | $1.7 million | 808 |
| Protect people from tobacco smoke (FCTC Art. 8) | Yes | -5% | 12,100 | $248 million | $4.4 million | 56 |
| Warning labels (FCTC Art. 11) | No † | | | | | |
| Plain packaging (FCTC Art. 11 Guidelines / Art. 11) | Yes | -4% | 10,100 | $207 million | $2.0 million | 102 |
| Bans on advertising (FCTC Art. 11/13) | No † | | | | | |
| Mass media campaigns (FCTC Art. 12) | No † | | | | | |
| Cessation support: brief advice to quit tobacco (FCTC Art. 14) | Yes | -2% | 3,800 | $75 million | $12.8 million | 6 |
| **Samoa** | **5 measures** | **-46%** | **950** | **$61 million** | **$5.9 million** | **10** |
| Raise cigarette taxes (FCTC Art. 6) | Yes | -17% | 300 | $22 million | $1.1 million | 20 |
| Protect people from tobacco smoke (FCTC Art. 8) | Yes | -13% | 300 | $20 million | $1.1 million | 18 |
| Warning labels (FCTC Art. 11) | No † | | | | | |
| Plain packaging (FCTC Art. 11 Guidelines / Art. 11) | Yes | -4% | 100 | $6 million | $620,000 | 10 |
| Bans on advertising (FCTC Art. 11/13) | Yes | -12% | 300 | $18 million | $640,000 | 28 |
| Mass media campaigns (FCTC Art. 12) | Yes | -11% | 300 | $17 million | $1.8 million | 9 |
| Cessation support: brief advice to quit tobacco (FCTC Art. 14) | No | | | | | |

| FCTC Tobacco Control Measure | Included in FCTC Tobacco Control Investment Case | Relative Change in Adult Smoking Prevalence Over 15 Years * | Tobacco-Related Deaths Averted Over 15 Years | Socioeconomic Losses Averted (US$) Over 15 Years | Implementation Costs for Policy/Intervention (US$) Over 15 Years | Return On Investment (ROI) for Policy/Intervention Over 15 Years |
|---|---|---|---|---|---|---|
| **Sri Lanka** | **6 measures** | **-40%** | **72,300** | **$3.6 billion** | **$20.8 million** | **172** |
| Raise cigarette taxes (FCTC Art. 6) | Yes | -14% | 24,100 | $1.2 billion | $1.6 million | 731 |
| Protect people from tobacco smoke (FCTC Art. 8) | Yes | -10% | 20,800 | $1.0 billion | $4.1 million | 247 |
| Warning labels (FCTC Art. 11) | No † | | | | | |
| Plain packaging (FCTC Art. 11 Guidelines / Art. 11) | Yes | -6% | 11,400 | $560 million | $2.0 million | 281 |
| Bans on advertising (FCTC Art. 11/13) | Yes | -7% | 15,000 | $739 million | $2.0 million | 369 |
| Mass media campaigns (FCTC Art. 12) | Yes | -12% | 23,800 | $1.2 billion | $8.1 million | 145 |
| Cessation support: brief advice to quit tobacco (FCTC Art. 14) | No | | | | | |
| Ban single stick cigarettes § | Yes | -5% | 10,500 | $516 million | $5.5 million | 94 |
| **Tunisia** | **7 measures** | **-46%** | **55,500** | **$2.3 billion** | **$35.9 million** | **63** |
| Raise cigarette taxes (FCTC Art. 6) | Yes | -25% | 30,100 | $1.2 billion | $2.4 million | 502 |
| Protect people from tobacco smoke (FCTC Art. 8) | Yes | -9% | 13,000 | $529 million | $4.8 million | 110 |
| Warning labels (FCTC Art. 11) | Yes | -7% | 9,900 | $400 million | $2.4 million | 164 |
| Plain packaging (FCTC Art. 11 Guidelines / Art. 11) | Yes | -2% | 3,300 | $136 million | $2.4 million | 55 |
| Bans on advertising (FCTC Art. 11/13) | Yes | -8% | 10,900 | $441 million | $2.5 million | 179 |
| Mass media campaigns (FCTC Art. 12) | Yes | -4% | 5,600 | $229 million | $3.9 million | 58 |
| Cessation support: brief advice to quit tobacco (FCTC Art. 14) | Yes | -1% | 1,000 | $38 million | $13.8 million | 3 |
| **Zambia** | **6 measures** | **-64%** | **40,300** | **$1.0 billion** | **$24.6 million** | **42** |
| Raise cigarette taxes (FCTC Art. 6) | Yes | -20% | 13,400 | $343 million | $2.4 million | 141 |
| Protect people from tobacco smoke (FCTC Art. 8) | Yes | -9% | 7,000 | $179 million | $5.4 million | 33 |
| Warning labels (FCTC Art. 11) | Yes | -16% | 11,400 | $292 million | $2.7 million | 109 |
| Plain packaging (FCTC Art. 11 Guidelines / Art. 11) | Yes | -6% | 4,400 | $113 million | $2.7 million | 42 |
| Bans on advertising (FCTC Art. 11/13) | Yes | -27% | 19,100 | $491 million | $2.8 million | 175 |
| Mass media campaigns (FCTC Art. 12) | Yes | -15% | 11,000 | $283 million | $4.7 million | 61 |
| Cessation support: brief advice to quit tobacco (FCTC Art. 14) | No | | | | | |

| FCTC Tobacco Control Measure | Included in FCTC Tobacco Control Investment Case | Relative Change in Adult Smoking Prevalence Over 15 Years * | Tobacco-Related Deaths Averted Over 15 Years | Socioeconomic Losses Averted (US$) Over 15 Years | Implementation Costs for Policy/Intervention (US$) Over 15 Years | Return On Investment (ROI) for Policy/Intervention Over 15 Years |
|---|---|---|---|---|---|---|
| **Upper-Middle-Income Countries** | | | | | | |
| **Armenia** | **7 measures** | **-52%** | **23,200** | **$1.9 billion** | **$22.7 million** | **85** |
| Raise cigarette taxes (FCTC Art. 6) | Yes | -22% | 7,400 | $589 million | $1.5 million | 381 |
| Protect people from tobacco smoke (FCTC Art. 8) | Yes | -8% | 4,600 | $384 million | $1.8 million | 215 |
| Warning labels (FCTC Art. 11) | Yes | -6% | 3,000 | $245 million | $840,000 | 291 |
| Plain packaging (FCTC Art. 11 Guidelines / Art. 11) | Yes | -2% | 1,000 | $83 million | $1.8 million | 46 |
| Bans on advertising (FCTC Art. 11/13) | Yes | -17% | 10,500 | $881 million | $1.0 million | 883 |
| Mass media campaigns (FCTC Art. 12) | Yes | -8% | 4,700 | $389 million | $7.0 million | 56 |
| Cessation support: brief advice to quit tobacco (FCTC Art. 14) | Yes | -4% | 1,400 | $108 million | $8.4 million | 13 |
| **Colombia** | **4 measures** | **-50%** | **154,500** | **$17.9 billion** | **$57.7 million** | **310** |
| Raise cigarette taxes (FCTC Art. 6) | Yes | -32% | 98,800 | $11.4 billion | $17.0 million | 669 |
| Protect people from tobacco smoke (FCTC Art. 8) | Yes | -10% | 34,200 | $3.9 billion | $15.0 million | 262 |
| Warning labels (FCTC Art. 11) | Yes | -12% | 42,300 | $4.9 billion | $7.3 million | 670 |
| Plain packaging (FCTC Art. 11 Guidelines / Art. 11) | Yes | -8% | 28,600 | $3.3 billion | $7.3 million | 452 |
| Bans on advertising (FCTC Art. 11/13) | No † | | | | | |
| Mass media campaigns (FCTC Art. 12) | No † | | | | | |
| Cessation support: brief advice to quit tobacco (FCTC Art. 14) | No | | | | | |
| **Costa Rica** | **4 measures** | **-35%** | **7,900** | **$1.4 billion** | **$12.1 million** | **118** |
| Raise cigarette taxes (FCTC Art. 6) | Yes | -12% | 2,700 | $478 million | $2.4 million | 199 |
| Protect people from tobacco smoke (FCTC Art. 8) | Yes | -8% | 2,100 | $379 million | $3.5 million | 109 |
| Warning labels (FCTC Art. 11) | No † | | | | | |
| Plain packaging (FCTC Art. 11 Guidelines / Art. 11) | Yes | -4% | 1,100 | $192 million | $1.8 million | 104 |
| Bans on advertising (FCTC Art. 11/13) | Yes | -14% | 3,400 | $628 million | $1.9 million | 331 |
| Mass media campaigns (FCTC Art. 12) | No † | | | | | |
| Cessation support: brief advice to quit tobacco (FCTC Art. 14) | No | | | | | |

| FCTC Tobacco Control Measure | Included in FCTC Tobacco Control Investment Case | Relative Change in Adult Smoking Prevalence Over 15 Years * | Tobacco-Related Deaths Averted Over 15 Years | Socioeconomic Losses Averted (US$) Over 15 Years | Implementation Costs for Policy/Intervention (US$) Over 15 Years | Return On Investment (ROI) for Policy/Intervention Over 15 Years |
|---|---|---|---|---|---|---|
| **Georgia** | **4 measures** | **-45%** | **53,100** | **$1.5 billion** | **$4.1 million** | **364** |
| Raise cigarette taxes (FCTC Art. 6) | Yes | -27% | 34,700 | $922 million | $1.1 million | 869 |
| Protect people from tobacco smoke (FCTC Art. 8) | Yes | -6% | 14,200 | $274 million | $1.5 million | 186 |
| Warning labels (FCTC Art. 11) | Yes | -9% | 16,100 | $338 million | $770,000 | 439 |
| Plain packaging (FCTC Art. 11 Guidelines / Art. 11) | No | | | | | |
| Bans on advertising (FCTC Art. 11/13) | Yes | -12% | 20,700 | $487 million | $780,000 | 622 |
| Mass media campaigns (FCTC Art. 12) | No | | | | | |
| Cessation support: brief advice to quit tobacco (FCTC Art. 14) | No | | | | | |
| **Jordan** | **6 measures** | **-57%** | **47,600** | **$10.1 billion** | **$40.8 million** | **247** |
| Raise cigarette taxes (FCTC Art. 6) | Yes | -39% | 34,000 | $7.2 billion | $4.7 million | 1,547 |
| Protect people from tobacco smoke (FCTC Art. 8) | Yes | -8% | 7,600 | $1.6 billion | $8.9 million | 180 |
| Warning labels (FCTC Art. 11) | Yes | -8% | 7,200 | $1.5 billion | $4.7 million | 324 |
| Plain packaging (FCTC Art. 11 Guidelines / Art. 11) | Yes | -4% | 3,600 | $764 million | $4.7 million | 164 |
| Bans on advertising (FCTC Art. 11/13) | Yes | -10% | 9,500 | $2.0 billion | $4.7 million | 423 |
| Mass media campaigns (FCTC Art. 12) | Yes | -4% | 4,100 | $862 million | $6.6 million | 130 |
| Cessation support: brief advice to quit tobacco (FCTC Art. 14) | No | | | | | |
| **Suriname** | **5 measures** | **-36%** | **1,770** | **$137 million** | **$10.0 million** | **14** |
| Raise cigarette taxes (FCTC Art. 6) | Yes | -13% | 600 | $47 million | $1.5 million | 31 |
| Protect people from tobacco smoke (FCTC Art. 8) | Yes | -6% | 400 | $29 million | $1.3 million | 23 |
| Warning labels (FCTC Art. 11) | No † | | | | | |
| Plain packaging (FCTC Art. 11 Guidelines / Art. 11) | Yes | -4% | 200 | $18 million | $740,000 | 25 |
| Bans on advertising (FCTC Art. 11/13) | No † | | | | | |
| Mass media campaigns (FCTC Art. 12) | Yes | -15% | 900 | $67 million | $1.2 million | 58 |
| Cessation support: brief advice to quit tobacco (FCTC Art. 14) | Yes | -2% | 100 | $7 million | $2.9 million | 2 |

All currency values are expressed as real, inflation-adjusted, 2021 US$.

* To consider the impact of multiple measures operating together, the investment cases applied constant proportional reductions following methods previously established in Levy (2007). The result of this is that estimated impact of the entire package of tobacco demand-reduction measures included for each country is less than the sum of estimated effect sizes for individual tobacco demand-reduction measures.

† Already fully implemented.

‡ Already mostly implemented.

§ Not an FCTC tobacco control measure. Custom analysis conducted for a single country as part of their FCTC tobacco control investment case.
